# A NEW MEDICATION-RELATED CUMULATIVE ANTICHOLINERGIC EXPOSURE INDEX EVALUATING RISK OF COGNITIVE IMPAIRMENT

**DOI:** 10.1093/geroni/igae098.3304

**Published:** 2024-12-31

**Authors:** Veronique Michaud, Kaveena Autar, Gerald Condon, Chelsea Honore, Maria Grisales, James Hickman, Jacques Turgeon

**Affiliations:** GalenusRx Inc, Orlando, Florida, United States; Hesperos, Clermont, Florida, United States; GalenusRx Inc, Orlando, Florida, United States; Hesperos, Orlando, Florida, United States; Hesperos, Orlando, Florida, United States; Hesperos, Orlando, Florida, United States; GalenusRx Inc, Orlando, Florida, United States

## Abstract

Given the associations between anticholinergic exposure and poor clinical outcomes in older adults, precise quantification of the cumulative anticholinergic burden appears advisable to evaluate the risk–benefit ratio of prescribing or deprescribing some medications. Numerous examples of anticholinergic quantification scales have been used for the past two decades. These were all developed using varying methods of measuring the anticholinergic activity of drugs and varying methods of classifying drugs into distinct potency categories. Recently, we have performed a systematic review of scales and models used to quantify anticholinergic burden in older adults. We have compiled the most comprehensive list of drugs with anticholinergic activities (N=642 drugs); the AntiCholinergic and Sedative Burden Catalog (ACSBC). Using human-on-a-chip technology, we have computed a quantitative Potency AntiCholinergic Score (PACS) by performing a dose-response LTP- suppression for each drug. Furthermore, to better inform our proprietary Aggregated PolyPharmacy Regimen Assessment Index for Safety and Equity (APPRAISE™), we have developed a new unique Medication-Related Cumulative Anticholinergic Exposure (MeReCAE) index. Using drug regimen data from 12,383 patients extracted from the Medical Expenditure Panel Survey (MEPS), we have sequentially proceeded to the virtual addition of amitriptyline, amantadine, asenapine and caffeine to the drug regimen of these patients. A more important shift toward higher MeReCAE values was observed when amitriptyline and asenapine were added to the drug regimen of patients. The quantitative analysis performed allowed us to determine exactly how many patients were in the Low, Moderate and High risk of the MeReCAE index following the addition of the tested drugs.

